# The Open-Air Treatment of Phthisis in England

**Published:** 1898-03-12

**Authors:** 


### THE OPEN-AIR TREATMENT OF PHTHISIS IN ENGLAND.

An interesting paper by Dr. Burton-Fanning is published in the *Lancet*, giving details of recent experience of the open-air treatment of phthisis as carried out at Cromer, where a convalescent home has been erected for the use of the patients of the Norfolk and Norwich Hospital. Since the spring of 1895 six patients at a time have been under treatment there.

The home stands 250 ft. above, and a quarter of a mile from the sea. There, in a shelter provided with moveable glass and wood walls which can be placed according to the wind, the patients sit in the open air. They adjourn to the shelter at half-past eight every morning, and pass the rest of the day there in long chairs. As a rule, they come indoors at sunset, but on very dry nights, both winter and summer, they have remained out of doors till ten.

Of course, they are well wrapped up, and great use is made of sacks thickly lined with wadding, into which they put their legs, and, if required, a hot-water bottle. Moderate evening fever (below 102 deg. F.) has been considered no contra-indication to following the treatment in its entirety.

